# Association of rs10204525 genotype GG and rs2227982 CC combination in programmed cell death 1 with hepatitis B virus infection risk

**DOI:** 10.1097/MD.0000000000016972

**Published:** 2019-08-30

**Authors:** Chunhong Huang, Tiantian Ge, Caixia Xia, Wei Zhu, Lichen Xu, Yunyun Wang, Fengtian Wu, Feifei Liu, Min Zheng, Zhi Chen

**Affiliations:** a State Key Laboratory for Diagnosis and Treatment of Infectious, Collaborative Innovation Center for Diagnosis and Treatment of Infectious Disease, The First Affiliated Hospital; b Department of Infectious Diseases, Affiliated Hangzhou First People's Hospital; c Key Laboratory of Precision Diagnosis and Treatment for Hepatobiliary and Pancreatic Tumor of Zhejiang Province, First Affiliated Hospital, Zhejiang University School of Medicine, Hangzhou, China.

**Keywords:** HBV, PD-1, single nucleotide polymorphism, susceptibility

## Abstract

Single nuclear polymorphism (SNP) of programmed cell death 1 (PD-1) was reported associated with hepatitis B virus (HBV) infection, but the SNP sites studied were limited. Whether the combination of 2 or more SNP sites could better represent the relationship between PD-1 SNP and HBV infection was not studied.

Eight hundred ninety-eight HBV-infected patients (222 asymptomatic carriers [AsC], 276 chronic hepatitis B, 105 acute-on-chronic liver failure, and 295 liver cirrhosis) and 364 health controls of South China were enrolled in this study. Four PD-1 SNPs (rs10204525, rs2227982, rs41386349, and rs36084323) were selected and detected by TaqMan probe. The frequency of allele, genotype, and combination of different SNPs were compared between different groups.

For allele frequency analysis, G allele of rs10204525 was protective factor (odds ratio (OR) = 0.823, 95% confidence interval (CI) = 0.679–0.997, *P* = .046) and T allele of rs2227982 was predisposing factor (OR = 1.231, 95% CI = 1.036–1.463, *P* = .018) in HBV infection. When analyzed in genotype frequency, the genotype GG of rs10204525 and CC of rs2227982 were protective factor of HBV infection. Combination of rs10204525 GG and rs2227982 CC was potent protective factor of HBV infection (OR = 0.552, 95% CI = 0.356–0.857, *P* = .007) and was also associated with lower HBV load (OR = 0.201, 95% CI = 0.056–0.728, *P* = .008) in AsC. The 4 SNP sites were not associated with progression of HBV-related liver disease.

Rs10204525 and rs2227982 of PD-1 associate with HBV infection and combination of the 2 SNP sites can better predict host susceptibility in HBV infection.

## Abstract 2

Supplemental Digital Content is available in the text

## 1 Introduction

Hepatitis B is one of the most common infectious diseases worldwide.^[1]^ Data from World Health Organization (WHO) website shows that 240 million individuals are infected with hepatitis B virus (HBV) (WHO 2016; http://www.who.int/mediacentre/factsheets/fs204/en/), and over 0.68 million death every year due to complications of hepatitis B, such as cirrhosis and liver cancer.^[2]^ It is a multistage process from asymptomatic carrier (AsC) to chronic hepatitis B (CHB) and even cirrhosis. Researches reveal that host's immune system, especially virus-specific T cells, plays an important role during the process.^[3,4]^

Programmed cell death 1 (PD-1) is known as co-inhibitory regulator of T-cell responses. T cells with high PD-1 expression are in exhaustion with decreased cytotoxic function, lower interferon-γ secretion, and reduced proliferating potential.^[5]^ HBV-specific T cells in CHB patients exhibit higher PD-1 expression, in comparison with healthy individuals, which was associated with impaired virus clearance.^[6,7]^ PD-1 blockage is capable to restore T cell functions and promote antiviral immunity.^[5,8,9]^

Host genetic component is one of the key factors that lead to persistent HBV infection.^[10]^ Variants of host genes, such as mannose-binding protein, estrogen receptor alpha, and human leukocyte antigen-DR affect the process of hepatitis B and HBV clearance.^[11–13]^ Single nucleotide polymorphism (SNP) is a kind of genetic variant which is the mutation in individual bases scattered throughout the genome. SNP influence the protein function that was encoded by the same gene. As PD-1 is important immune regulatory molecules in HBV infection,^[6,7]^ its genetic polymorphism may influence outcome of HBV infection.

Although the association of genetic polymorphism of PD-1 with HBV has been investigated previously,^[14–16]^ the SNP sites they analyzed were limited and more other sites were waiting to be tested. What's more, most investigations only studied the SNP sites separately. As each SNP sites had its own influence, the combination of them may be more powerful in elucidating the relationship between genetic factor of PD-1 and HBV infection. Also, the researches mainly focused on the influences on HBV associated cancer and the influence on acute-on-chronic liver failure (ACLF) was not reported. To further clarify the relationship between PD-1 polymorphism and HBV infection, we selected 4 PD-1 SNPs (rs10204525, rs2227982, rs41386349, rs36084323), and performed a retrospective case–control study.

## 2 Material and methods

### 2.1 Ethics statement

The study is approved by the ethics committee of Zhejiang University, China. The written informed consent is obtained from each participant. The study is carried out in accordance with the guidelines of the 1975 Declaration of Helsinki.

### 2.2 Study participants

This study recruited 898 HBV-infected patients at the First Affiliated Hospital, Zhejiang University from March, 2015 to January, 2016 and 364 health controls (HCs) from the physical examination center of the hospital during the time. Patients with positive serum hepatitis B surface antigen (HBsAg) or HBV DNA were diagnosed as HBV infection and were classified into 4 groups according to the Guideline of Prevention and Treatment for CHB^[17]^: 222 AsC, 276 CHB, 295 liver cirrhosis (LC), and 105 ACLF. AsC was diagnosed according to the criteria described previously.^[18]^ CHB was diagnosed as seropositive HBsAg or HBV DNA, with consistently or recurrently high level of alanine transaminase (ALT) for more than 6 months. LC was diagnosed if any sign of cirrhosis was found by imaging examination. ACLF was diagnosed if serum total bilirubin (TBIL) was higher than 10 times of normal upper limit (171 μM) or increases more than 17.1 μM/d, and prothrombin time activity ≤40% (or international normalized ratio ≥1.5).

The patients were excluded with the following diseases:

(1) hepato-carcinoma;
(2) other hepatitis virus infections;
(3) other diseases which can lead to immune system disorder such as auto-immune diseases and cancer;
(4) major organ dysfunction like heart, lung.

**Figure 1:**
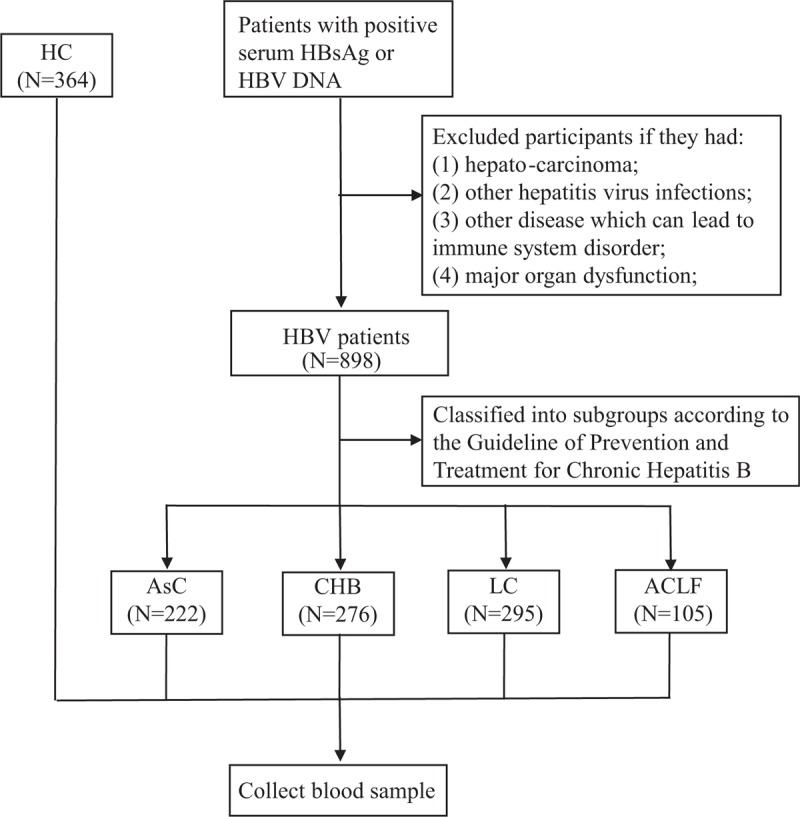
Study participants included and flow chart of study design. Patients with positive serum HBsAg or HBV infection was regarded as HBV infection and included. After excluded unqualified patients according to the exclusion criteria, 898 participants were finally included and classified into 4 sub-groups: 222 AsC, 276 CHB, 295 LC, and 105 ACLF. 364 health controls were also included. ACLF = acute-on-chronic liver failure; AsC = asymptomatic carriers, CHB = chronic hepatitis B, HBV = hepatitis B virus, LC = liver cirrhosis.

The blood sample (approximately 2 mL) was collected from the filtered participants. All the procedure above was shown in Figure 1.

### 2.3 DNA extraction

Whole blood samples were collected into ethylene diamine tetraacetic acid coated tubes and stored at −80°C. Genomic DNA was extracted from whole blood using QIAamp DNA Blood Mini Kit (Qiagen, Valencia, CA) according to the manufacturer's instruction and then stored at −80°C.

### 2.4 SNP selection and genotyping

Candidate SNP sites of PD-1 were collected to HapMap Chinese population data. The SNPs with a minor allele frequency (MAF) >0.01 were included. Finally, 4 PD-1 SNP sites were included in this study. The details of the SNP sites were shown in Table 1. The SNPs were genotyped by TaqMan probe (Applied Biosystems, Foster City).

**Table 1:**
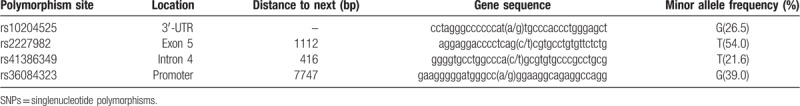
Locations and allele frequencies of SNPs.

### 2.5 Statistical analysis

At first, Hardy–Weinberg equilibrium (HWE) was tested for each SNP by Pearson *χ*^2^ test. Then, we compared the distribution of demographic characteristics of each SNP between different groups. For analysis of allele and genotype, the Pearson *χ*^2^ test (or Fisher exact test) were used. Multi-factor logistic regression analysis was selected if the 2 groups have different distribution of age and sex. When comparing the genotype frequency of each SNP between different groups, we used the following gene models: (if “A” was major allele and “a” was minor) dominant gene model (Aa + aa vs AA), recessive gene model (aa vs AA + Aa), and additive gene model (aa vs Aa vs AA), codominant gene model (aa vs AA and Aa vs AA). All the statistical analyses were carried out using SPSS 20.0 software (SPSS Inc., Chicago, IL). The difference between 2 groups was considered statistically significant if *P* < .05 (2-sided). Odds ratios (ORs) and 95% confidence intervals (CIs) were used to show the degree of association between groups.

## 3 Results

### 3.1 Clinical features of the study participants

The demographics and clinical characteristics of HCs, HBV patients and its subgroups including AsC, CHB, ACLF, and LC, were shown in Table 2. The average age was 42.37 ± 12.44 in the HBV group and was 40.81 ± 13.76 in HCs. The HBV patients included 637 (70.94%) males and 261 (29.06%) females. The HCs consisted of 235 (64.56%) males and 129 (35.44%) females. No significant differences were found in age and gender between HBV patients and controls (*P* > .05, data not shown). But the distribution of age and gender in the 4 subgroups was not equal (*P* < .05, data not shown). ALT, aspartate aminotransferase, TBIL, and albumin in different groups changed, as they indicating the liver function which changed with the disease progression.

**Table 2:**
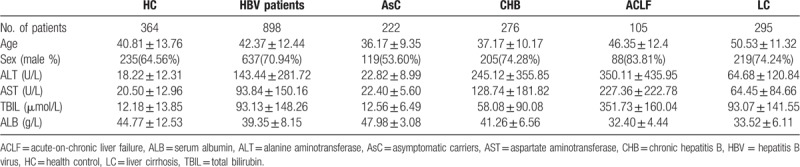
Clinical demographics of the groups.

### 3.2 Genotypic and allele distribution

The genotypic and allele distribution of the 4 SNP sites were shown in Table S1. The MAFs of 4 PD-1 SNPs were as follows: rs10204525, 0.265; rs2227982, 0.540; rs41386349, 0.216 (Table 1). The observed values of every SNP genotypes showed no significant differences when compared with expected values in HCs (all *P* > .05, Table S2), therefore the genotypes distribution of the 4 SNPs was in HWE.

### 3.3 Rs10204525 and rs2227982 polymorphisms were associated with HBV infection

The results of association between PD-1 polymorphisms and HBV susceptibility were shown in Table 3. In the 4 PD-1 SNPs, rs10204525 and rs2227982 polymorphisms were associated with HBV infection. For allele frequency, the minor allele G of rs10204525 was protective factor and T of rs2227982 predisposing factor with HBV susceptibility (OR = 0.823, 95% CI = 0.679–0.997, *P* = .046; OR = 1.231, 95% CI = 1.036–1.463, *P* = .018, respectively).

**Table 3:**
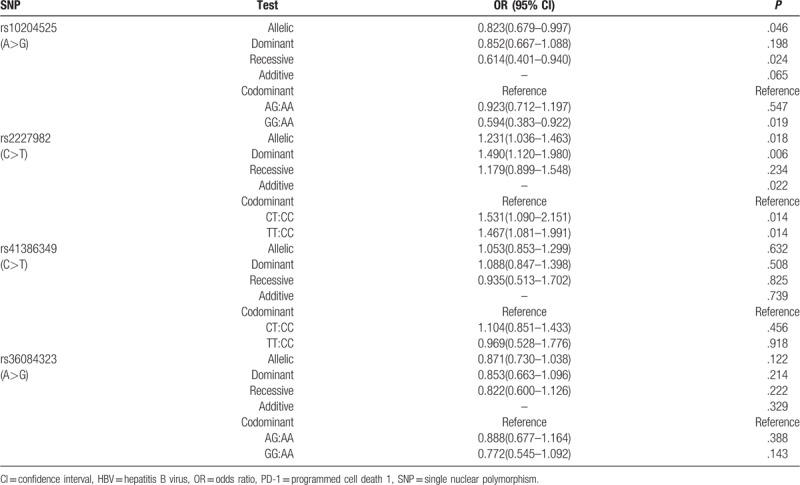
Genotype and allele distribution of PD-1 SNPs in HBV patients and health controls.

Then the genotype was analyzed. For rs10204525, subjects carrying genotype GG had a significantly decreased risk of getting infected with HBV (recessive gene model: OR = 0.614, 95% CI = 0.401–0.940, *P* = .024; codominant gene model (GG:AA): OR = 0.594, 95% CI = 0.383–0.922, *P* = .019). For rs2227982, the TT and CT frequency of HBV patients was significantly higher than that of HCs (dominant gene model: OR = 1.490, 95% CI = 1.120–1.980, *P* = .006; codominant gene model (TT:CC): OR = 1.467, 95% CI = 1.081–1.991, *P* = .014; codominant gene model (CT:CC): OR = 1.531, 95% CI = 1.090–2.151, *P* = .014). From all the results above, the genotype GG of rs10204525 and CC of rs2227982 were protective factor of HBV infection.

The polymorphisms of rs41386349 and rs36084323 were not related to HBV infection. The allele frequency of minor allele T of rs41386349 and G of rs36084323 in HBV patients showed no significant difference compared with HC (OR = 1.053, 95% CI = 0.853–1.299, *P* = .623; OR = 0.871, 95% CI (0.730–1.038), *P* = .122, respectively). As for the genotype frequency, 2 polymorphism sites still showed no significant between HBV patients and HC (all *P* > .05).

### 3.4 Combination of rs10204525 GG and rs2227982 CC was associated with lower HBV infection risk

As rs10204525 G and rs2227982 C were both protective factors of HBV infection, we tried to analyze whether their combination could cooperate in defending HBV infection. As shown in Table 3, recessive model could well represent the difference between wild-type and mutation of rs10204525 on HBV infection, so we divided the 3 genotypes AA AG and GG into AA + AG group and GG group. Genotypes of rs2227982 were divided into CC group and CT + TT group, following the same rule. As a result, there were 4 combinations of the groups between the 2 SNPs, as shown in Table S3, which also displayed the distributions of the 4 combinations in different subject groups.

Combination of rs10204525 AA + AG and rs2227982 CC and combination of rs10204525 GG and rs2227982 TT/CT had the same distribution between HBV patients and HC, compared with rs10204525 AA + AG and rs2227982 TT/CT (*P* = .097 and *P* = .457, respectively) (Table 4). Combination of rs10204525 GG and rs2227982 CC showed significantly lower frequency in HBV patients (OR = 0.552, 95% CI = 0.356–0.857, *P* = .007), which meant this combination was associated with lower HBV infection risk.

**Table 4:**
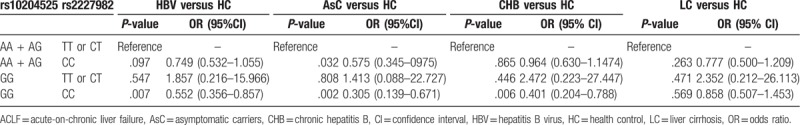
Combination analysis of rs10204525 and rs2227982.

When dividing HBV patients into subgroups, we found that combination of rs10204525 GG and rs2227982 CC showed coordinated lower frequency in AsC (OR = 0.305, 95% CI = 0.139–0.671, *P* = .002), CHB (OR = 0.401, 95% CI = 0.204–0.788, *P* = .006) but not LC (OR = 0.858, 95% CI = 0.507–1.453, *P* = .569) compared with HC.

### 3.5 Combination of rs10204525 GG and rs2227982 CC were associated with lower HBV load in AsC

Subjects of AsC were classified into 2 group, HBV undetectable and HBV detectable. Allele and genotype analysis of rs10204525 and rs227982 were carried out in AsC about the influence on HBV load and the correlation was poor (Table S4). When the combination was taken into account, combination of rs10204525 GG and rs2227982 CC had lower percentage in HBV detectable group (OR = 0.201, 95% CI = 0.056–0.728, *P* = .008) (Table 5).

**Table 5:**
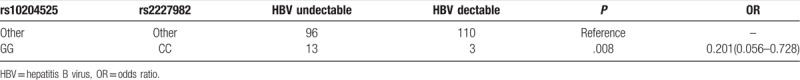
People with genotype GG(rs10204525) and CC(rs2227982) had higher proportion of HBV undetected in AsC subjects.

### 3.6 Association of PD-1 polymorphisms with disease progression

We performed the following 3 comparisons: CHB versus AsC, LC versus CHB, and ACLF versus CHB, to unveil the relation between PD-1 variants and progression of HBV-related liver diseases (Table 6). LC had higher rs10204525 G allele compared with CHB (*P* = .033). T allele of rs41386349 showed lower frequency in CHB compared with AsC (*P* = .042). As for comparison of rs41386349 genotype between LC versus CHB, dominant model analysis displayed that TT was protective factor of LC (OR = 0.605, 95% CI = 0.410–0.891, *P* = .011), but when in codominant model, CT showed predisposing factor on LC (OR = 2.495, 95% CI = 1.020–5.784, *P* = .045) and TT had the same frequency with CC (*P* = .166).

**Table 6:**
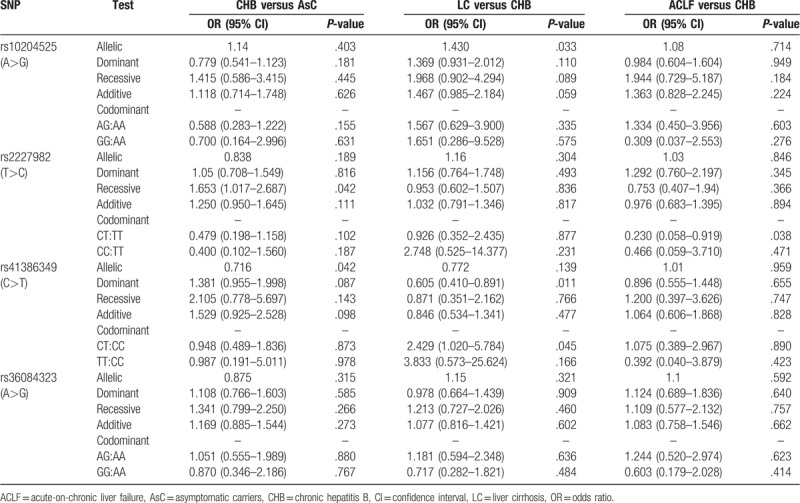
Genotype and allele distribution of PD-1 SNPs between different subgroups.

## 4 Discussion

In this study, we found T allele of rs2227982 was suggested to be a predisposition factor of HBV infection. Consistent with previous studies, G allele on rs10204525 site was a protective factor in HBV infection.^[15,16]^ We also proved that combination of rs10204525 GG with rs2227982 CC had better protection from HBV infection and the combination also related with a lower HBV load in AsC. In all, the results above further show that genetic variants of PD-1 play roles in HBV infection and combination of SNP sites are more efficient in defending HBV infection. The results contribute to illustrating the way host genetic factors influence HBV infection and provide a method of combining SNPs together to predict the risk of HBV infection and outcome.

Statistics data on the WHO website (http://www.who.int/mediacentre/factsheets/fs204/en/) shows that there is high prevalence in East Asian with a 5% to 10% CHB in adult population and only less than 1% of Western Europe and North America is chronically infected. Brazilian families of Asian origin have a higher occurrence of HBsAg (*P* < .0001) than of Western origin,^[19]^ which meant ethnic factors may influence HBV infection. According to data on the 1000 Genomes and HapMap, the frequency of protective rs10204525 G is much lower in Chinese (0.267 for Han Chinese in Beijing and 0.879 for British in England and Scotland) and the predisposing factor rs2227982 T is higher (0.495 for Han Chinese in Beijing and 0.006 for British in England and Scotland). These may explain why China has a high rate of HBV infection in host genetic aspect.

T cells with higher PD-1 expression are in exhaustion and blocking PD-1 can restore it and promote antiviral immunity.^[5,8,9]^ It has also been reported that a genetic variant (rs11568821) may affect PD-1 mRNA level by change the binding affinity of RUNX (a transcriptional factor of PD-1).^[20]^ Combining these previous conclusions with our work, we believe that the genetic variants, rs2227982 and rs10204525 of PD-1 influent patients’ HBV susceptibility and disease progression by regulating the expression and function of PD-1.

Rs2227982, which was associated with the risk and disease progression of ankylosing spondylitis and breast cancer in previous reports,^[21,22]^ was revealed to be relevant to HBV susceptibility in our study. Rs2227982 locates in the 5th exon of PD-1 and its polymorphism leads to a nonsynonymous mutation and results in an amino acid substitution (Ala to Val) during protein synthesis, which may influence the function of PD-1.

G allele of rs10204525 was a protective factor in HBV infection in our study, which was consistent with the previous work from Shaanxi Province, China.^[15,16]^ Data from the Beijing Genomics Institute revealed that population immunity was different between the North and the South.^[23]^ Considering their work was conducted on the people of Northwest China and our work studied the people on the Eastern China, rs10204525 could be used as a nationwide protective marker of HBV infection.

Other researches focused on the relationship between rs10204525 and PD-1 expression found out G of rs10204525 associated with lower PD-1 expression. PD-1 mRNA levels in peripheral blood nuclear cells of CHB were sequentially decreased from rs10204525 genotypes AA, AG to GG.^[24]^ Subjects with genotype AA of PD-1 rs10204525 had higher PD-1 expressions in tumor tissues, peritumor tissues, and cirrhotic tissues than with other genotypes.^[25]^ With lower inhibitory PD-1 expression, the immune system may show greater anti-virus function, and the individual may not easily get infected. Rs10204525 GG genotype altered tumor necrosis factor-α (TNF-α) to higher levels in HBV patients compared with controls.^[26]^ As TNF-α has potent anti-virus function,^[27]^ this may serve another way to explain why G allele of rs10204525 is a protective factor in HBV infection.

As 1 SNP site could not well represent the influence of PD-1 genetic variants in HBV infection, we analyzed the genotype combination of rs10204525 and rs2227982. Results revealed that the combination of rs10204525 GG and rs2227982 CC were more efficient in reducing HBV infection risk and HBV load compared with GG or CC alone. This may show a way by integrating different SNP sites to fully elucidate the susceptibility of HBV infection from host genetic perspective. The integration also provide evidence for the doctor in individually treating HBV patients.

Still, more works are ongoing to be done. First, the population tested was limited to Eastern China, and more subjects from other populations should be included to verify the result. Second, we have revealed the potential association between the selected SNPs and HBV infection at this step, on the next step, we will explore the molecular mechanism underling the relationship, especially on rs2227982. Third, we can select a series of representative SNPs on genes which influence susceptibility and progression of HBV and make a rating scale to evaluate individual's outcome on getting HBV infection from the host perspective.

In conclusion, this study showed that rs10204525 and rs2227982 polymorphisms of PD-1 affect host's susceptibility to HBV infection. Combination of rs10204525 GG and rs2227982 CC can better predict host susceptibility in HBV infection, which is also associated with lower HBV load.

## Author contributions

**Data curation:** Chunhong Huang, Tiantian Ge, Caixia Xia, Feifei Liu.

**Formal analysis:** Chunhong Huang, Caixia Xia, Fengtian Wu.

**Investigation:** Caixia Xia.

**Methodology:** Chunhong Huang, Zhi Chen.

**Project administration:** Chunhong Huang.

**Resources:** Wei Zhu.

**Software:** Lichen Xu, Yunyun Wang.

**Supervision:** Min Zheng, Zhi Chen.

**Writing – original draft:** Chunhong Huang.

**Writing – review and editing:** Chunhong Huang, Zhi Chen.

Zhi Chen orcid: 0000-0002-0848-1502.

## Supplementary Material

Supplemental Digital Content
