# Supplementary material for: Association of rs10204525 genotype GG and rs2227982 CC combination in programmed cell death 1 with hepatitis B virus infection risk
Source: Medicine (Baltimore). 2019 Aug 30;98(35):e16972. doi: 10.1097/MD.0000000000016972 (PMC6736136; doi:10.1097/MD.0000000000016972)
Supplement: Supplemental Digital Content [file medi-98-e16972-s001.docx]

Table S1. Distribution of PD-1 polymorphisms in all groups

|  | Genotype | HC(N=364) | AsC(N=222) | CHB(N=276) | ACLF(N=105) | LC(N=295) |
| --- | --- | --- | --- | --- | --- | --- |
| rs10204525 | AA | 189 | 118 | 164 | 64 | 156 |
| (A>G) | AG | 137 | 95 | 98 | 33 | 110 |
|  | GG | 38 | 9 | 14 | 8 | 29 |
| Allele | A | 515 | 331 | 426 | 161 | 422 |
|  | G | 213 | 113 | 126 | 49 | 168 |
| rs2227982 | CC | 96 | 32 | 58 | 18 | 66 |
| (C>T) | CT | 170 | 120 | 133 | 58 | 141 |
|  | TT | 98 | 70 | 85 | 29 | 88 |
| Allele | C | 362 | 184 | 249 | 94 | 273 |
|  | T | 366 | 260 | 303 | 116 | 317 |
| rs36084323 | AA | 133 | 92 | 108 | 40 | 122 |
| (A>G) | AG | 161 | 100 | 124 | 48 | 117 |
|  | GG | 70 | 30 | 44 | 17 | 56 |
| Allele | A | 427 | 284 | 340 | 128 | 361 |
|  | G | 301 | 160 | 212 | 82 | 229 |
| rs41386349 | CC | 227 | 139 | 150 | 59 | 194 |
| (C>T) | CT | 121 | 77 | 112 | 41 | 89 |
|  | TT | 16 | 6 | 14 | 5 | 12 |
| Allele | C | 575 | 355 | 412 | 159 | 477 |
|  | T | 153 | 89 | 140 | 51 | 113 |

Abbreviations: HC, health control; AsC, asymptomatic carriers; CHB, chronic hepatitis B; ACLF acute-on-chronic liver failure; LC, liver cirrhosis

Table S2. Hardy-Weinberg calculations for the four SNPs in health control

| SNP | Genetype1(obs\|exp) | Genetype2(obs\|exp) | Genetype3(obs\|exp) | chisquare | pvalue |
| --- | --- | --- | --- | --- | --- |
| rs10204525 | AG(137\|150.68) | AA(189\|182.16) | GG(38\|31.16) | 3.000 | 0.083 |
| rs2227982 | CC(96\|90.00) | TT(98\|92.00) | CT(170\|182.00) | 1.581 | 0.209 |
| rs36084323 | AG(161\|176.55) | GG(70\|62.23) | AA(133\|125.23) | 2.823 | 0.093 |
| rs41386349 | CC(227\|227.08) | TT(16\|16.08) | CT(121\|120.85) | 0.001 | 0.980 |

Table S3. Distribution of different genotype combination of rs10204525 and rs2227982 in all groups.

| rs10204525 | rs2227982 | HC | HBV infection | AsC | CHB | LC |
| --- | --- | --- | --- | --- | --- | --- |
| AA+AG | TT+CT | 267 | 719 | 189 | 216 | 227 |
| AA+AG | CC | 59 | 119 | 24 | 46 | 39 |
| GG | TT+CT | 1 | 5 | 1 | 2 | 2 |
| GG | CC | 37 | 55 | 8 | 12 | 27 |

Abbreviations: HC, health control; AsC, asymptomatic carriers; CHB, chronic hepatitis B; ACLF acute-on-chronic liver failure; LC, liver cirrhosis.

Table S4. Genotype and allele distribution of PD-1 SNPs between HBV detectable and HBV undetectable group in AsC

| SNP | Test | OR(95%CI) | P |
| --- | --- | --- | --- |
| rs10204525 | Allelic | 0.819(0.529-1.267) | 0.369 |
| (A>G) | Dominant | 0.842(0.492-1.443) | 0.532 |
|  | Resessive | 0.432(0.105-1.774) | 0.232 |
|  | Additive | - | 0.458 |
|  | Codominant | reference | reference |
|  | AG:AA | 0.902(0.519-1.569) | 0.715 |
|  | GG:AA | 0.413(0.098-1.731) | 0.214 |
| rs2227982 | Allelic | 1.284(0.873-1.888) | 0.204 |
| （C>T） | Dominant | 1.534(0.719-3.269) | 0.266 |
|  | Resessive | 1.360(0.763-2.426) | 0.296 |
|  | Additive | - | 0.405 |
|  | Codominant | reference | reference |
|  | CT:CC | 1.405(0.638-3.095) | 0.398 |
|  | TT:CC | 1.773(0.761-4.134) | 0.183 |
